# CASP5 and CR1 as potential biomarkers for Kawasaki disease: an Integrated Bioinformatics-Experimental Study

**DOI:** 10.1186/s12887-021-03003-5

**Published:** 2021-12-11

**Authors:** Yazdan Rahmati, Hasan Mollanoori, Sajad Najafi, Sajjad Esmaeili, Mohammad Reza Alivand

**Affiliations:** 1 Department of Medical Genetics and Molecular Biology, Faculty of Medicine, Iran University of Medical Sciences, Tehran, Iran; 2 Student Research Committee, Department of Medical Biotechnology, School of Advanced Technologies in Medicine, Shahid Beheshti University of Medical Sciences, Tehran, Iran; 3 Medical Biology Research Center, Health Technology Institute, Kermanshah University of Medical Sciences, Kermanshah, Iran; 4 Department of Medical Genetics, Faculty of Medicine, Tabriz University of Medical Sciences, Tabriz, Iran

**Keywords:** Gene expression, Hub genes, RT-PCR, ROC curve, WGCNA

## Abstract

**Background:**

Kawasaki disease (KD) is a pediatric inflammatory disorder causes coronary artery complications. The disease overlapping manifestations with a set of symptomatically like diseases such as bacterial and viral infections, juvenile idiopathic arthritis, Henoch-Schönlein purpura, infection of unknown etiology, group-A streptococcal and adenoviral infections, and incomplete KD could lead to misdiagnosis of the disease.

**Methods:**

In the present study, we applied weighted gene co-expression network analysis (WGCNA) to identify network modules of co-expressed genes in GSE73464 and also, limma package was used to identify the differentially expressed genes (DEGs) in KD expression arrays composed of GSE73464, GSE18606, GSE109351, and GSE68004. By merging the results of WGCNA and limma, we detected hub genes. Then, analyzed the peripheral blood mononuclear cells (PBMCs) of 16 patients and 8 control subjects using Real-Time Polymerase Chain Reaction (RT-PCR) to evaluate the previous results.

**Results:**

We assessed the diagnostic potency of the screened genes by plotting the area under curve (AUC). We finally identified 2 genes *CASP5(Caspase 5)* and *CR1*(Complement C3b/C4b Receptor 1) which were shown to potentially discriminate KD from other similar diseases and also from healthy people.

**Conclusions:**

The results of RT-PCR and AUC confirmed the diagnostic potentials of two suggested biomarkers for KD.

**Supplementary Information:**

The online version contains supplementary material available at 10.1186/s12887-021-03003-5.

## Background

Kawasaki Disease (KD) is a rare systemic vasculitis disorder which affects the medium and small arteries particularly coronary arteries in infants and young children [1]. The disease is diagnosed globally with the highest incidence reported for the eastern Asia countries including, in order of magnitude, Japan, South Korea, and Taiwan; in non-Asian countries, however, substantial and meaningful differences in the incidence rate have been demonstrated [1–4]. Generally, the difference has been linked with the racial composition of the societies [5]. A functional single-nucleotide polymorphism (itpkc-3)in the inositol 1,4,5-trisphosphate 3-kinase C (ITPKC) gene has been associated with enhanced susceptibility to the disease and developing coronary artery aneurisms, so that C allele of *itpkc-3* gene can conduce to immune hyper-reactivity in KD (e.g., by increase in interleukin-2 [IL-2] transcript level) [6]. Nonetheless, the etiology of KD remains enigmatic [5–7]; accordingly, it is required to further explore the molecular mechanisms of the disease to achieve a decisive diagnosis.

The clinical manifestations of KD include a series of symptoms such as fever lasting longer than five days, bilateral non-purulent conjunctivitis and cervical lymphadenopathy, rashes, lip fissures, erythema and edema in oral mucosa (characteristic strawberry tongue) and peripheral extremities, and etc. which are similar to those of other types of neonatal illnesses [8] such as epistaxis, juvenile idiopathic arthritis, scarlet fever, and etc., and this can lead to misdiagnosis of the condition [9]. Delayed or missed diagnosis can pose the patient at higher risk of coronary artery abnormalities [10]. till now, a large number of biomarkers including those designed for inflammatory, proteomics, gene expression profiles, and micro-RNA characteristics have been failed in diagnostic approaches due to unacceptable sensitivity and specificity [10–13], and so, they may couldn’t suggest confirmation in the diagnostic decision [14]. Therefore, introduction of a novel, decisive, prognostic, or diagnostic biomarker would be an inevitable necessity for the KD as a complicated condition requiring immediate diagnosis. The genetic basis known for KD has been interesting for the researchers and clinicians to develop a genetic approach for diagnosis and prediction of prognosis in patients with KD. The genetic approaches might be classified into ‘candidate gene approaches’ and ‘genome-wide approaches’ [14–17]. Chaudhary et al. [16] have enlisted the studies conducted on the genetic markers of KD. They, however, have stressed on irreproducibility of the results among different nations. So, one drawback may be concluded from the insufficient genes list is that it doesn’t make possible to accurately diagnose and predict prognosis of the disease.

Over the past decade, substantial pathogenetic clues have been found for various diseases such as immune reactions via the genome-wide approach [18, 19]. In this regard, using the DNA microarray method, alteration in expression levels including up- and down-regulation of thousands of genes and also pathological mechanisms can be detected in a single chip such as experiments conducted for KD [20]. Weighted gene co-expression network analysis (WGCNA), likewise, empowers the scientists to explore network alterations and basic mechanisms among highly correlated genes and to also help find new biomarkers from disease associated genes cluster [21–23].

In the present study, we investigated the co-expressed genes in KD patients using WGCNA package to explore network modules. Then, four microarray datasets of KD from the Gene Expression Omnibus (GEO) repository were integrated to find the differentially expressed genes (DEGs) in the patient’ samples compared to control groups. By merging the results, we screened 35 genes and then through evaluation of their aberrant expression with symptomatically like diseases including bacterial and viral infections, JIA (juvenile idiopathic arthritis), HSP (Henoch-Schönlein purpura), infection of unknown etiology, GAS (group A streptococcal) infection, human adenovirus (HAdV) infection, and incomplete KD, two genes were selected as potential biomarkers. Eventually, we employed Real-Time Polymerase Chain Reaction (RT-PCR) to substantiate the selected genes.

## Methods

### Data preprocessing and weighted co-expression network construction

Following search of “Kawasaki disease” and “*Homo sapiens*” keywords at https://www.ncbi.nlm.nih.gov/geo, GEO series GSE73464 was selected for weighted co-expression construction. To normalize the dataset, we used quantile normalization method in limma (linear models for microarray data) R package [24] and log2 transformation. An only single measure was included for each gene by the aggregate function in S4Vectors package. To construct the weighted gene co-expression network of GSE73464, we used WGCNA R package. Constructing the weighted gene network is necessarily accompanied by choosing the soft thresholding power β, which culminates in adjacency calculation through creating co-expression similarity. We conducted the analysis of the topology network and the improved selection of a proper soft-thresholding power using pickSoftThreshold function. The adjacency results were converted into Topological Overlap Matrix (TOM) to minimize the impacts of noise and spurious associations. The results of TOM and cutreeDynamic function act respectively as inputs and branch cutting to produce the gene-based dendrogram. The minimum module size was set at 30, and we used the module detection sensitivity deep Split 2 in blockwiseConsensusModules function to construct the network. To determine the module(s) containing the significant genes, we assessed: (1) the relationships of individual genes and clinical data by defining Gene Significance, and (2) the correlation between the eigengene module and gene expression profile for each module as a quantitative criterion of modular membership.

### Identification of clinically significant modules

We measured the relationships between individual genes and clinical data through defining Gene Significance (GS) as the absolute value. For each module, a quantitative criterion of module membership (MM) was considered the correlation between the eigengene module and gene expression profile through which similarity of all genes on the array was measured and determined to every module. By employment of GS and MM, it is feasible to identify interesting module(s) containing the genes with the great significance of both clinical data and module membership.

### Protein-Protein interaction (PPI) Network Construction

We constructed the protein-protein interaction (PPI) network of the selected module using STRING and cytoscape [25]. The cytoscape molecular complex detection (MCODE) plugin was used to identify the finest clusters [26]. ClueGO v2.5.3 [27] was utilized to perform pathway enrichment analysis of the genes, and the most important signaling pathways based upon KEGG database were detected [28].

### Expression array datasets

Three other expression arrays including GEO series GSE18606, GSE109351, and GSE68004 from the GEO (containing a total number of 104 control and 196 patient samples), then were normalized, and eventually DEGs were screened under the settings of a cutoff defined as *p*-value < 0.05 and |log2fold change| ≥ 1.

### Comparison of diagnostic values of detected hub genes in KD and symptomatically like diseases

We used the GSE73464 expression array dataset to find the gene distinctions between KD and a set of other symptomatically like diseases including bacterial and viral infections, JIA, HSP, and infection of unknown etiology, and also GSE68004 dataset was used to explore other distinctive genes between KD and another group of similar infections such as GAS, HAdV, and incomplete KD. The DEGs between KD and other diseases, and also compared to healthy controls (defined as log2 fold change >1 in KD vs. all the comparative groups) were, as well, regarded as biomarkers. To determine the diagnostic value of the hub genes, we plotted receiver operating characteristic (ROC) curve and the area under the curve (AUC). P-values of < 0.05 were considered for statistical significance.

### Experimental Methods

#### Subjects and sample preparation

Blood samples were collected in the tubes containing EDTA from the children referred to Ali-Asgar Hospital of Iran University of medical sciences, Tehran, Iran diagnosed with KD. Regarding the approved codes of Ethics Committee form the Iran University of Medical Sciences as well as the declaration of Helsinki, the written informed consent forms were signed for each sampling. The participants included 32 patients and 16 healthy children aged less than five years old, out of patients 62.5% (20 subjects) were male and remaining 37.5% (12 subjects) were female.

#### Peripheral blood mononuclear cell (PBMC) Isolation

Each blood sample was diluted with the phosphate buffered saline (PBS) in 1:1 ratio. After adding 3 ml of ficoll solution, the tubes were centrifuged at 400 g for 30 min. Then the layer containing peripheral blood mononuclear cells (PBMCs) was separated from other blood cell components, washed thrice with PBS buffer to achieve higher purity, and eventually centrifuged again at 200 g for 10 min.

#### RNA extraction and cDNA production

Total RNA was extracted from the PBMCs in each group by Total RNA Miniprep Purification Kit (Cat 17,200, GeneMark Diagnostics Inc., Georgia, and USA). The quality of extracted RNA was confirmed by measuring the optical density of OD_260_/OD_280_ ratio and viewing on 2% gel electrophoresis. Furthermore, concentration of the extracted RNA was measured using NanodropND-1000 spectrophotometer (Thermo Fisher Scientific, Wilmington, USA). Then we converted the RNA to cDNA using 2X RT-PCR Pre-Mix (Taq) Kit (BioFACT, South Korea).

#### Primer design

The primer’s sequences were designed using Genscript online tool (available at https://www.genscript.com/tools/real-time-pcr-tagman-primer-design-tool). To evaluate their quality, the primers were assessed on online oligonucleotide calculator tool (http://biotools.nubic.northwestern.edu/OligoCalc.html), and Gene Runner software (Hastings Software, Colorado, and USA). The primers are available in Supplementary Table 1.

#### Real-Time PCR

The lyophilized powder of the primers (Cinaclone, Iran) was dissolved by adding sterile distilled water to each tube (according to the manufacturer’s instructions); then were stored as stock at -20 °C. Step One RT-PCR system (Applied Biosystems, Forster City, CA, USA) and SYBR green PCR kit (Takara Biotech, China) were utilized to conduct the quantitative RT-PCR (qRT-PCR) for measuring the gene expression levels. The results were then normalized based on GAPDH expression levels. The final solution volume was 15 µL, comprised of 1 µl cDNA, 1 µl of each forward and reverse primers (2 µl in total), 10 µl Taq polymerase, and 2 µl DEPC water. A number of forty cycles were chosen to conduct the PCR under the following condition: 95 °C (2 min), 95 °C (30 s), and 60 °C (40 s). A negative control for each individual gene was provided at each time to examine the possible contaminations. Finally, the expression fold change of the examined genes was assessed using the Threshold Cycle (CT) method by the following formulae:

$$\text{R}={2}^{- (\varDelta \varDelta \text{C}\text{T})}$$

$$\varDelta \varDelta \text{C}\text{T}={(\text{C}{\text{T}}_{\text{t}\text{a}\text{r}\text{g}\text{e}\text{t}}-\text{C}{\text{T}}_{\text{r}\text{e}\text{f}\text{e}\text{r}\text{e}\text{n}\text{c}\text{e}})}_{\text{h}\text{e}\text{a}\text{l}\text{t}\text{h}\text{y}}-{(\text{C}{\text{T}}_{\text{t}\text{a}\text{r}\text{g}\text{e}\text{t}}-\text{C}{\text{T}}_{\text{r}\text{e}\text{f}\text{e}\text{r}\text{e}\text{n}\text{c}\text{e}})}_{\text{p}\text{a}\text{t}\text{i}\text{e}\text{n}\text{t}}$$

## Results

### WGCNA and Modules identification

**Fig. 1:**
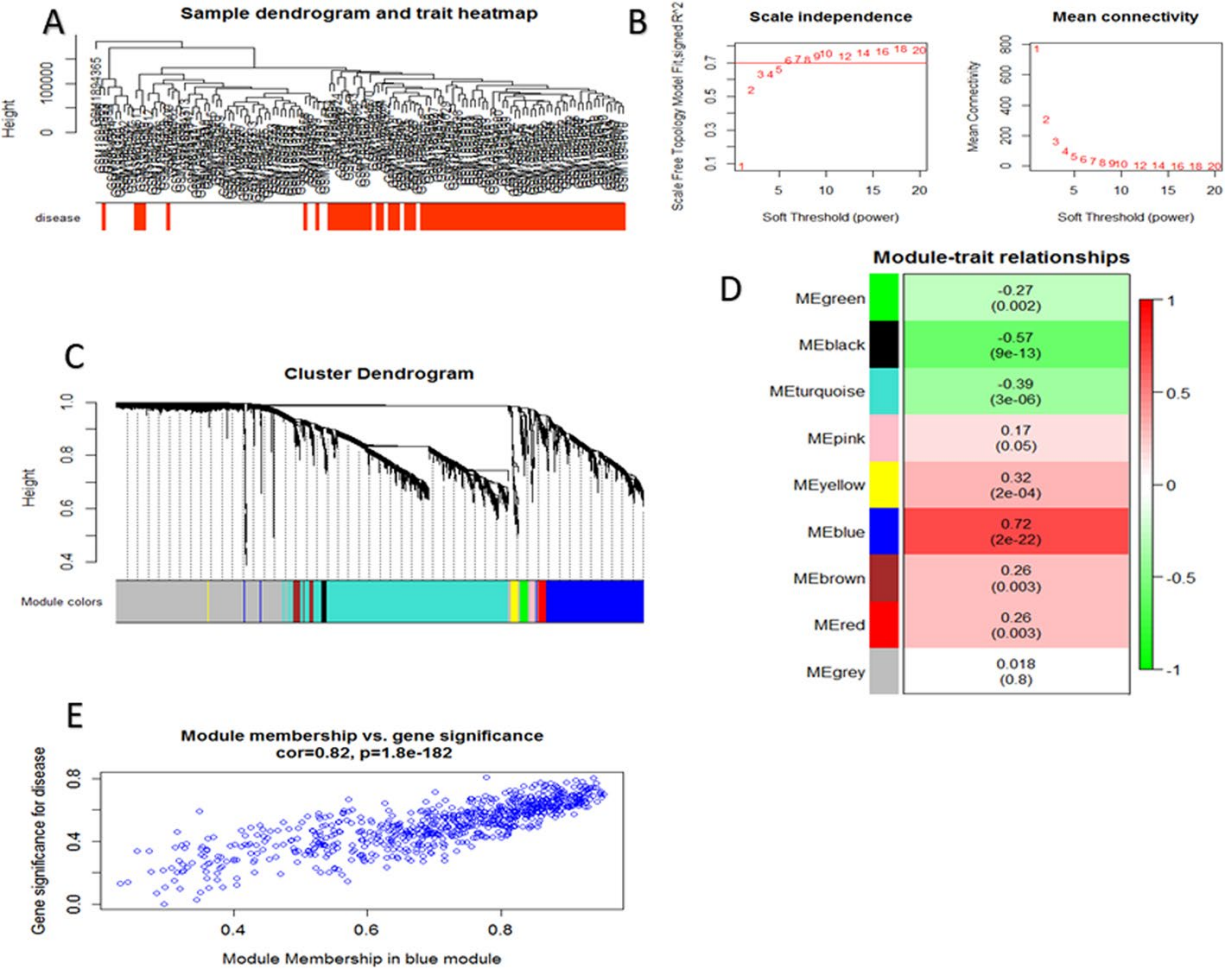
WGCNA data for GSE73464. (**A**) Clustering dendrogram of samples based on their Euclidean distance and how the clinical traits relate to the sample dendrogram, white means a low value, red a high value. (**B**) Analysis of network topology for various soft-thresholding powers. Scale-free fit index and the mean connectivity for various soft-thresholding powers. (**C**) Clustering dendrogram of DEGs with dissimilarity based on topological overlap, together with assigned module colors. Each color represents a module in the constructed gene co-expression network. (**D**) Module-trait associations for DEGs. Each row corresponds to a module eigengene, column to a trait. Each cell contains the corresponding correlation and p-value. The table is color-coded by correlation according to the color legend. (**E**) (**F**) scatterplot of Gene Significance (GS) for disease gene significance vs. Module Membership (MM) of blue module

We constructed the co-expression networks (modules) of GSE73464 dataset via WGCNA. The clinical trait and gene expression aberrations between the patient and control groups were thoroughly fit in the dendrogram of the samples and included in the expression dataset (Fig. 1 A). According to the approximate scale-free topology as the crucial criterion to opt the most precise soft thresholding power, we selected power 6 for the rest of analysis (Fig. 1B). Then, the clustering dendrogram of genes was plotted with a large minimum module size of 30 using the medium sensitivity (deep Split=2) for cluster splitting (Fig. 1 C). Clustering dendrogram of GSE73464 included nine modules (Fig. 1D). Among all created modules, blue (correlation =0.82) was selected and accentuated per dataset (Fig. 1E). Finally, the ultimate gene list containing 253 genes was achieved in blue module.

### Enrichment Analysis

**Fig. 2:**
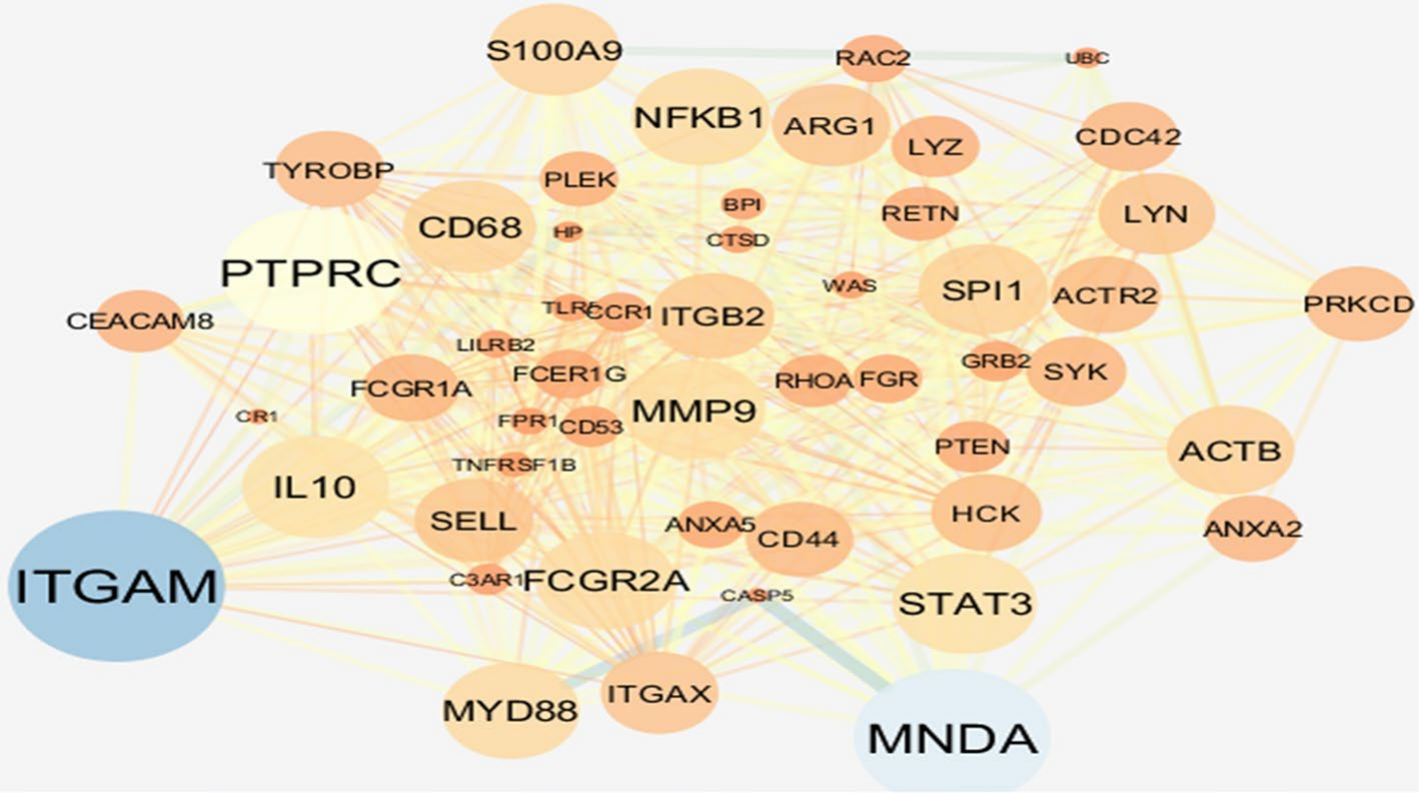
Protein-protein interaction network of the DEGs. Showing genes with the highest number of interactions. The gradual change in the size indicates the connectivity degree. The gradual change in the color indicates the betweenness centrality. The thickness of the edges stands for the credibility

**Fig. 3:**
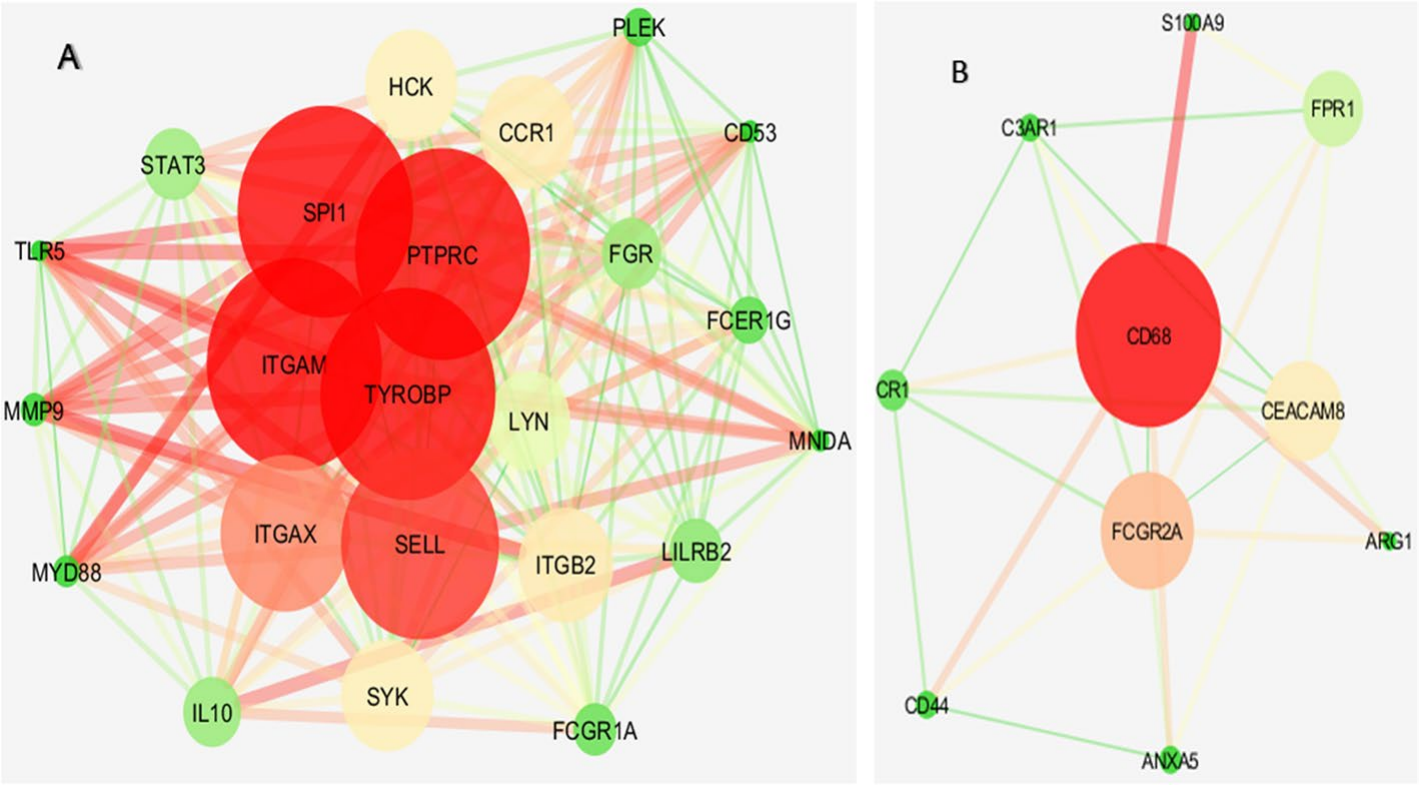
A total of 2 modules were identified in the PPI network using the MCODE tool in Cytoscape software. Module (**A**) had 23 nodes and 199 edges. Module (**B**) had 10 nodes and 26 edges

**Fig. 4:**
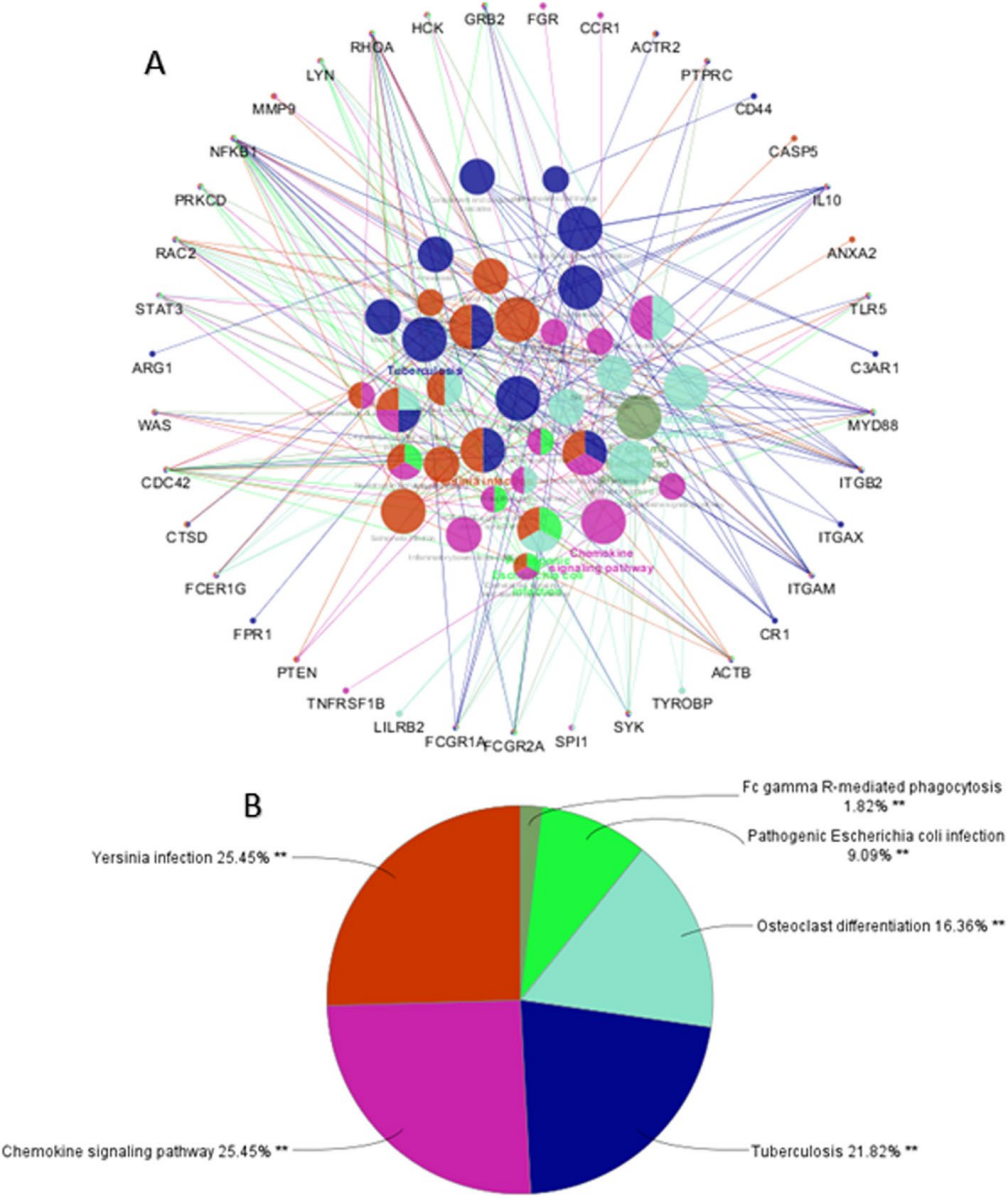
Enriched KEGG pathways using the ClueGo plugin of Cytoscape: (**A**) functionally grouped networks based on KEGG database of Genes with terms as nodes linked based on their κ score level (≥0.3). Only the most significant term in each group is presented. Associated proteins were visualized using CluePedia. (**B**) A chart with functional groups, including specific terms for the detected signaling pathways in the KD

A total number of253 genes were selected in the blue module for enrichment analysis and then their PPI network was constructed using the STRING database and cytoscape software. Among them, 50 genes were selected as hub genes through screening the top candidates with the highest number of interactions, Closeness and Betweenness Centrality (Fig. 2). According to the network sizes, MMP9, ITGB2, SPI1, SELL, and CD68 play an important role in pathogenesis of KD. We determined the interactive relationships between the hub genes in the network using the MCODE plugin. Two clusters were found with the following characteristics: 23 nodes and 199 edges in cluster 1, and 10 nodes and 26 edges in cluster 2. They were identified from the MCODE based on a scoring system (cutoff k-score = 12) (Fig. 3). The screened genes were enriched and analyzed through the Cytoscape plug-in ClueGo based on KEGG database. As it can be concluded from the pie chart, chemokine signaling pathway, tuberculosis, yersinia infection, pathogenic *Escherichia coli* infection, and fc gamma r-mediated phagocytosis are the most important signaling pathways (Fig. 4 A, and 4B).

### Identification of DEGs

**Fig. 5:**
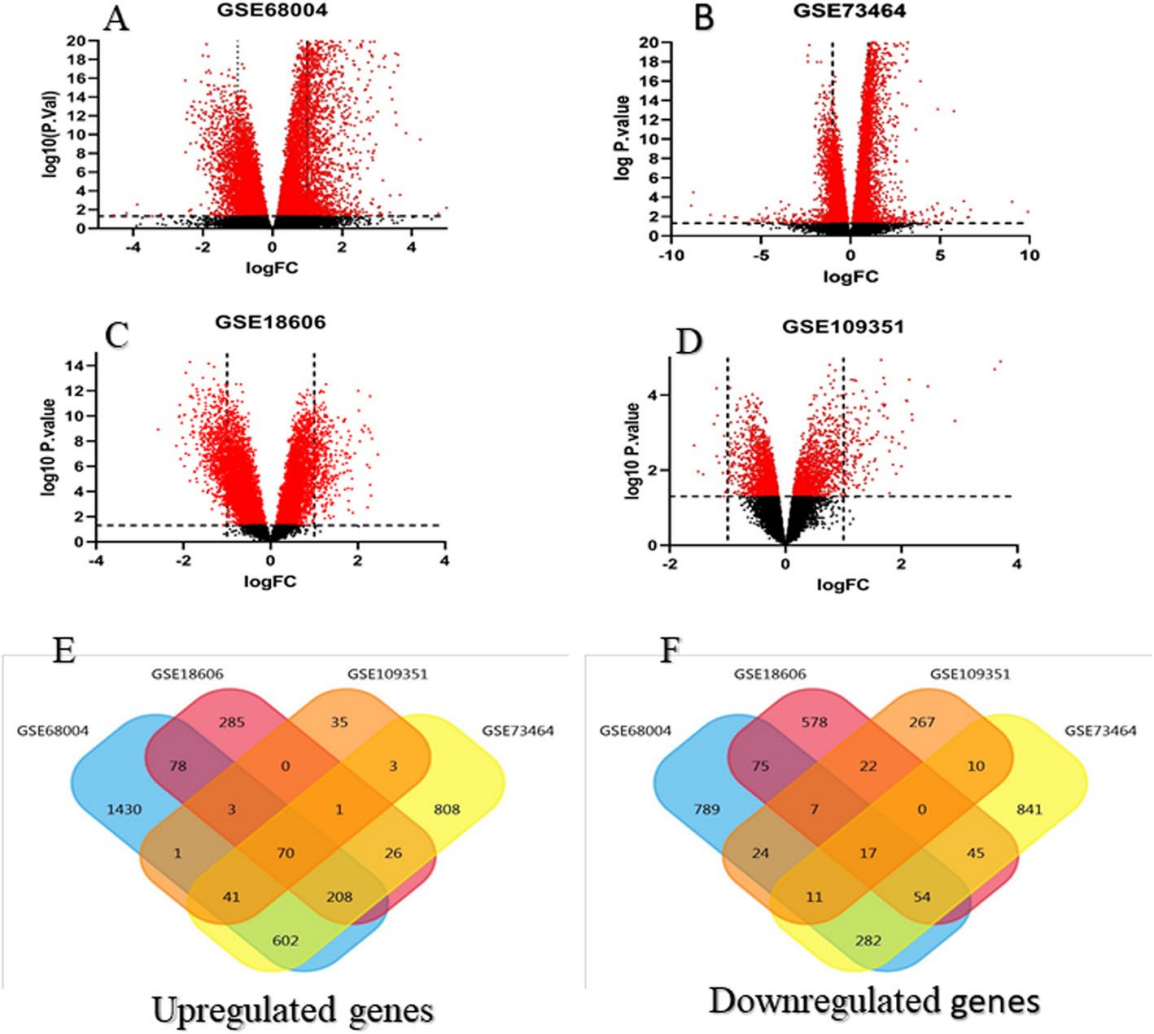
Differentially expressed genes and common differentially expressed genes in four datasets between KD and healthy control samples. (**A**, **B**, **C**, **D**) The volcano plots of differentially expressed genes in GSE68004, GSE73464, GSE18606, and GSE109351 respectively using pValue < 0.05 and |log2fold change| ≥ 1. (**E**, **F**) Common differentially expressed genes in four datasets

Four expression arrays (GSE73464, GSE18606, GSE109351, and GSE68004) were analyzed to evaluate the alterations of gene expression levels between healthy control and patient samples using Limma package. We screened all the DEGs using |log2FC| > 1 and *p*-value < 0.05 as the threshold and then were showed in volcano plots (Fig. 5 A-D). Following overlapping, we identified 70 upregulated and 17 downregulated common genes (Fig. 5E, F). Through merging the blue module with DEGs, 35 genes were identified that could discriminate KD from healthy controls.

### Biomarkers to differentiate KD from other symptomatic-overlapping febrile conditions

**Fig. 6:**
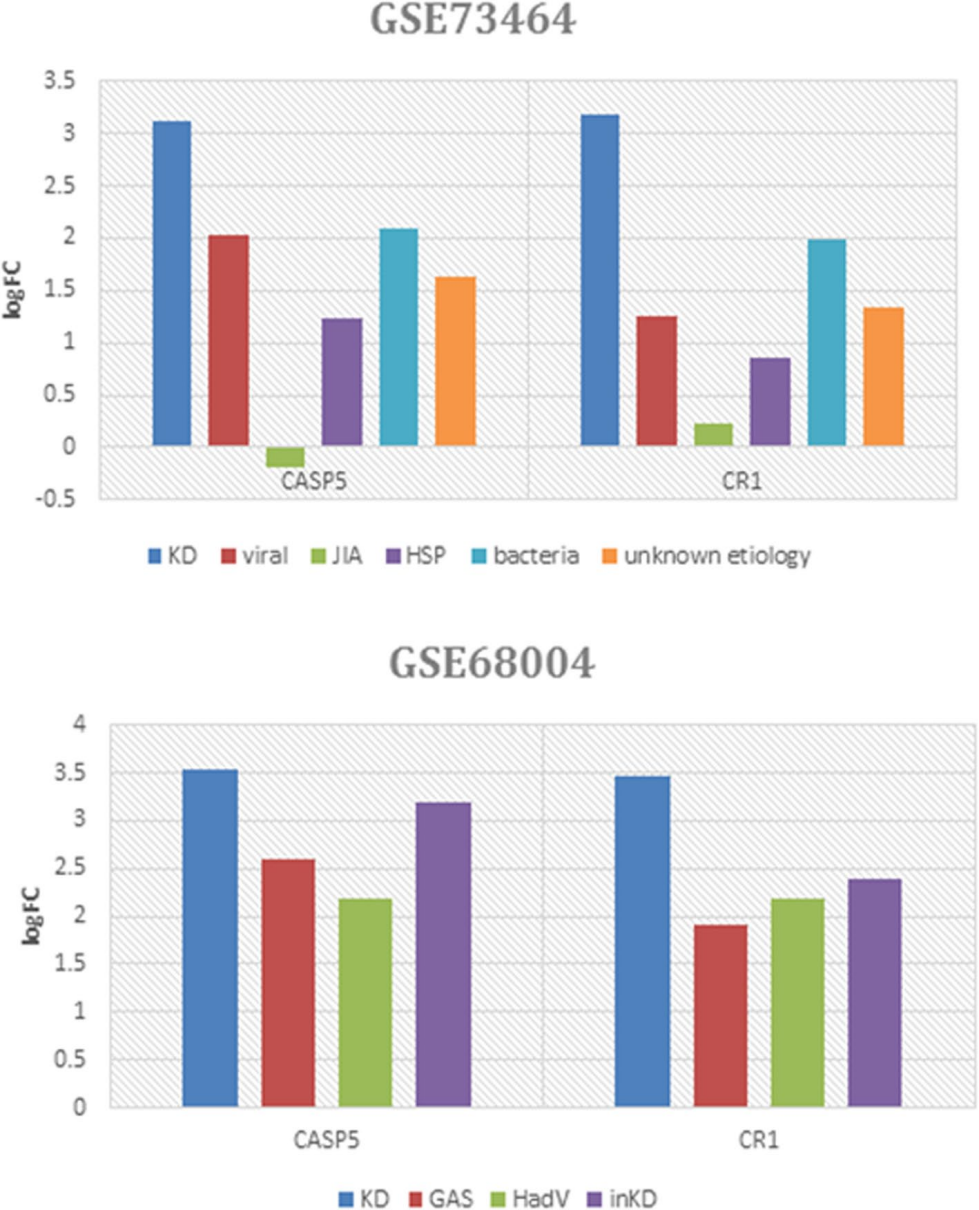
Expression level of CASP5 and CR1 in KD and other symptomatic-similar diseases in GSE73464 and GSE68004

To date, there is no definite DEG-based diagnostic test for KD. Accordingly, we included two expression array datasets (GSE73464, and GSE68004) in our investigation aimed to help distinguish KD from other resembling febrile conditions through discerning the most informative and distinctive gene expression signature. According to our criterion, by comparing the resemblance and dissimilarity of expressional patterns of our hub genes which were specified via their logFC values (defined as log2 fold change >1 in KD vs. all of the comparator groups), two genes signature comprised of*CASP5* and *CR1* were identified (Fig. 6).

### Real-Time PCR

**Fig. 7:**
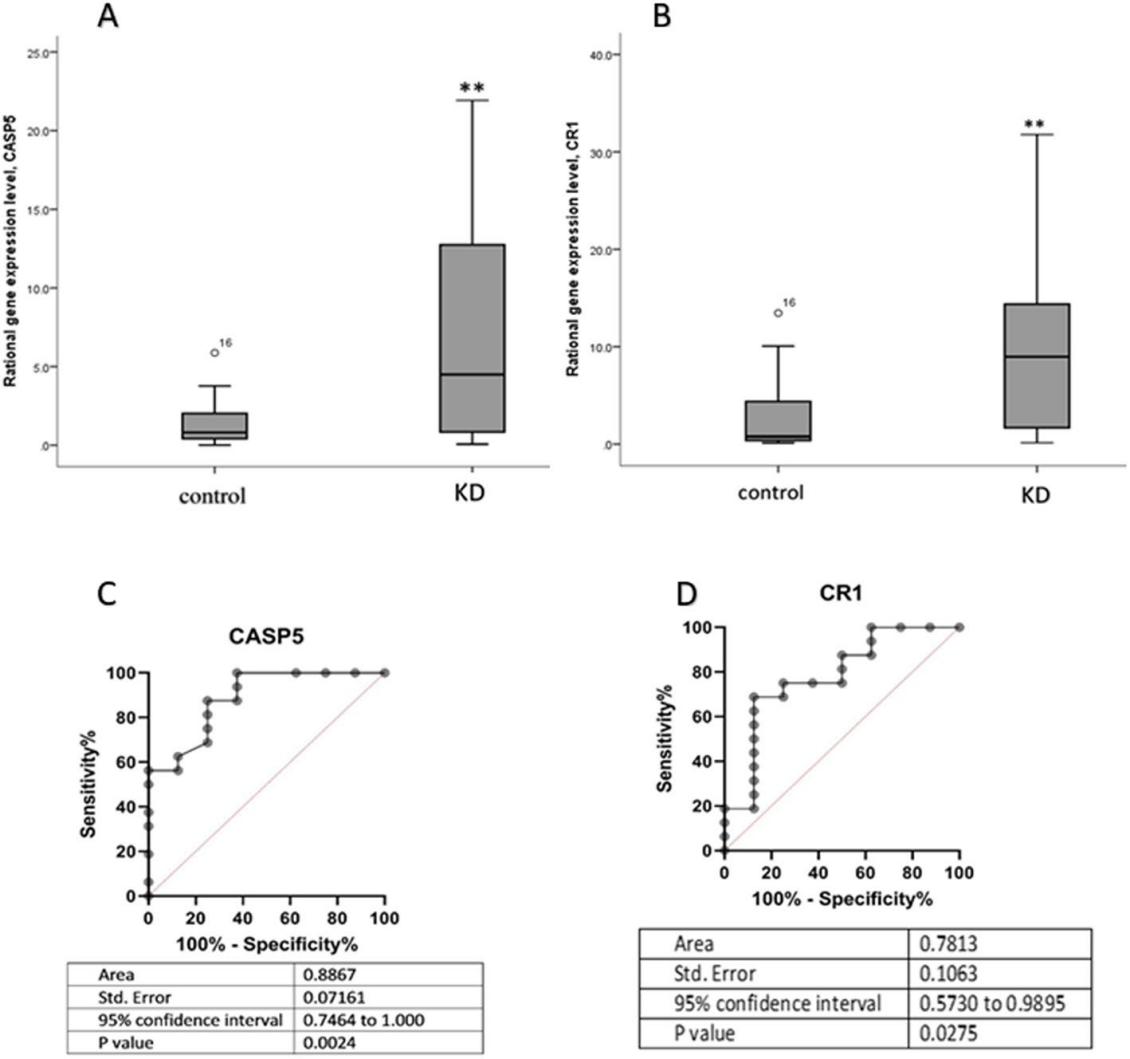
(**A**-**B**) Box plots of serum levels of CASP5 and CR1 in 16 KD patients compare to 8 control samples. (**C**-**D**) Receiver operating characteristic (ROC) curves of (**C**) CASP5, (**D**) CR1, based on real-time PCR results

To experimentally verify the differential expression of *CR1* and *CASP5* in KD patients, qRT-PCR assay was used, the results showed significantly increased expression in KD patients compared to healthy controls (Fig. 7 A, B). According to RT-PCR data, ROC curve analysis showed that both *CASP5* and *CR1* could distinguish KD from the common febrile diseases (Fig. 7 C, D).

### Hub genes validation

A ROC curve was generated to verify the diagnostic performance of *CASP5* and *CR1* based on the disease vs. healthy control expression array databases (GSE73464, GSE68004, GSE18606, and GSE109351). The AUC showed that *CASP5* and *CR1* indicated perfect diagnostic efficiency for KD compared to normal samples. The AUC of CASPS5 related to GEO series GSE73464, GSE68004, GSE18606, and GSE109351 was 0.949, 0.982, 0.955, and 1 respectively (Supplementary 1 A-D). Same measures of AUC for *CR1* were 0.866, 0.713, 0.883, and 1 respectively (Supplementary 2 A-D). AUC was also employed to evaluate the diagnostic power of *CASP5* and *CR1* to distinguish KD from other similar febrile conditions (Supplementary 3).

## Discussion

Despite more than fifty years since Dr. Tomisaku Kawasaki primarily introduced Kawasaki disease [29], no confirmatory test has been developed for the clinical diagnosis of KD. Moreover, the pathophysiological mechanisms of systemic vasculitis in the disease have not yet been completely understood. In histopathological assessments, it has been found that vasculitis, as an inflammatory cardiovascular disorder develops in KD, damages coronary arteries and cardiac sequelae leading to coronary failure and formation of aneurysms [30]. Genetically, as well, the genes included in the early detection might not be applied to differentiate KD from symptomatically similar diseases.

Wright et al. [31] introduced a signature composed of a set of genes, in order of priority regarding logistic regression coefficient, as specific biomarkers to distinguish KD from the similar diseases including CACNA1E, DDIAS, KLHL2, PYROXD2, SMOX, ZNF185, LINC02035, CLIC3, S100P, IFI27, HS.553,068, CD163, and RTN. The authors revealed that these biomarkers expressed differentially in samples taken from the patients with KD. Using a bio-signature of 13 transcripts in a parallel regularized regression model, Wright et al. [11] established their criteria for differential diagnosis as follows: the logFC >1 in KD compared to the control group, and logFC difference of at least ‘1’ when comparing KD to the similar diseases. Some uncertainties are seen in the criteria regarding the precise detection of the differential genes in KD especially if an almost identical gene expression pattern similar to KD’s condition obscures the experiment.

In the present study, comparing the expression levels of 35 mentioned genes in KD compared to other symptomatically like diseases mainly infections of bacterial or viral causes, juvenile idiopathic arthritis, Henoch-Schönlein purpura, infection of unknown etiology, group A streptococcal and adenoviral infections, and incomplete KD, we revealed two upregulated genes including *CR1* and *CASP5* in samples of patients with KD compared to other. Both the genes were found to be significantly over-expressed in KD patients (logFC >1).

The complement receptor type 1 (*CR1*) processes and clears opsonized complement safety complexes [32]. Several roles have been attributed to this protein including negative regulation of complement cascades, mediating the immune compliance and phagocytosis, and inhibiting both classical and alternative pathways of apoptosis. Significant upregulation of *CR1* in KD besides the role of *CR1* in immunity, for instance in innate immune response, might elucidate the inflammatory symptoms in [30].

Caspase-5 together with caspase-1 and caspase-4 plays role in apoptosis and contributes significantly to the immunity [33]. Caspase-1 expresses constitutively, whereas caspase-5 (the human orthologue of caspase-11) expression is induced by bacterial lipopolysaccharide (LPS) [34]. Caspase-5 is found in a limited number of tissues including peripheral blood lymphocytes, liver, placenta, spleen, and colon. Conversely, caspase-4 exists in all body tissues [35, 36]. Mutations in *CASP5* gene have been associated with several types of cancer such as leukemia, endometrial cancer, lung cancer, gastrointestinal tract and colorectal cancers [37]. As well, a direct relationship might exist between skin inflammation and *CASP5* upregulation [37]. Caspase-5 and caspase-1 may functionally synergize to regulate the processing of interleukin-1β (IL-1β), pro-IL18, and pyroptosis [38].

## Conclusion

Using a series of experiments aim to evaluate the expression level of genes in KD patients compared to healthy control samples and also between patient’s samples before and after treatment by intravenous immunoglobulin (IVIG), we identified two genes *CASP5* and *CR1* as specific biomarkers for KD. The results of our study suggest that KD could be distinguished from a group of diseases with similar manifestations by evaluating the expression level of the candidate genes. Designing a diagnostic test based on the gene expression signature could provide early diagnosis and therefore, help prescribe immediate treatment which is critical in KD patients and consequently can prevent life-threatening cardiac complications in the affected children.

## Supplementary Information

**Additional file 1.**

## Data Availability

The data that support the findings of this study are available on request from the corresponding author. The data are not publicly available due to privacy or ethical restrictions.

## Abbreviations

AUC: Area Under the Curve
CR1: Complement C3b/C4b Receptor 1
CASP5: Caspase 5
KD: Kawasaki Disease
ITPKC: 1,4,5-trisphosphate 3-kinase C
IL-2: interleukin-2
GEO: Gene Expression Omnibus
JIA: juvenile idiopathic arthritis
HSP: Henoch-Schönlein purpura
GAS infection: group A streptococcal infection
HAdV infection: human adenovirus infection
RT-PCR: Real-Time Polymerase Chain Reaction
limma R package: linear models for microarray data R package
MCODE: molecular complex detection
ROC curve: Receiver Operating Characteristic curve
IVIG: intravenous immunoglobulin
WGCNA: Weighted gene co-expression network analysis
