# Supplementary material for: CASP5 and CR1 as potential biomarkers for Kawasaki disease: an Integrated Bioinformatics-Experimental Study
Source: BMC Pediatr. 2021 Dec 11;21:566. doi: 10.1186/s12887-021-03003-5 (PMC8665509; doi:10.1186/s12887-021-03003-5)
Supplement: Supplementary file 1 — Additional file 1. [file 12887_2021_3003_MOESM1_ESM.docx]

Supplementary data

Table 1: Designed primers for real-time PCR

| Gene | Primers |
| --- | --- |
| CASP5 | Forward 5′-GGTGAAAAACATGGGGAACTC-3′  Reverse 5′-TGAAGAACAGAAAGCAATGAAGT-3′ |
| CR1 | Forward 5′ -TGGCATGGTGCATGTGATCA-3′  Reverse 5′ - TCAGGGCCTGGCACTTCACA-3′ |
| GAPDH | Forward 5′ - ACCACAGTCCATGCCATCAC-3′  Reverse 5′ - TCCACCACCCTGTTGCTGTA -3′ |


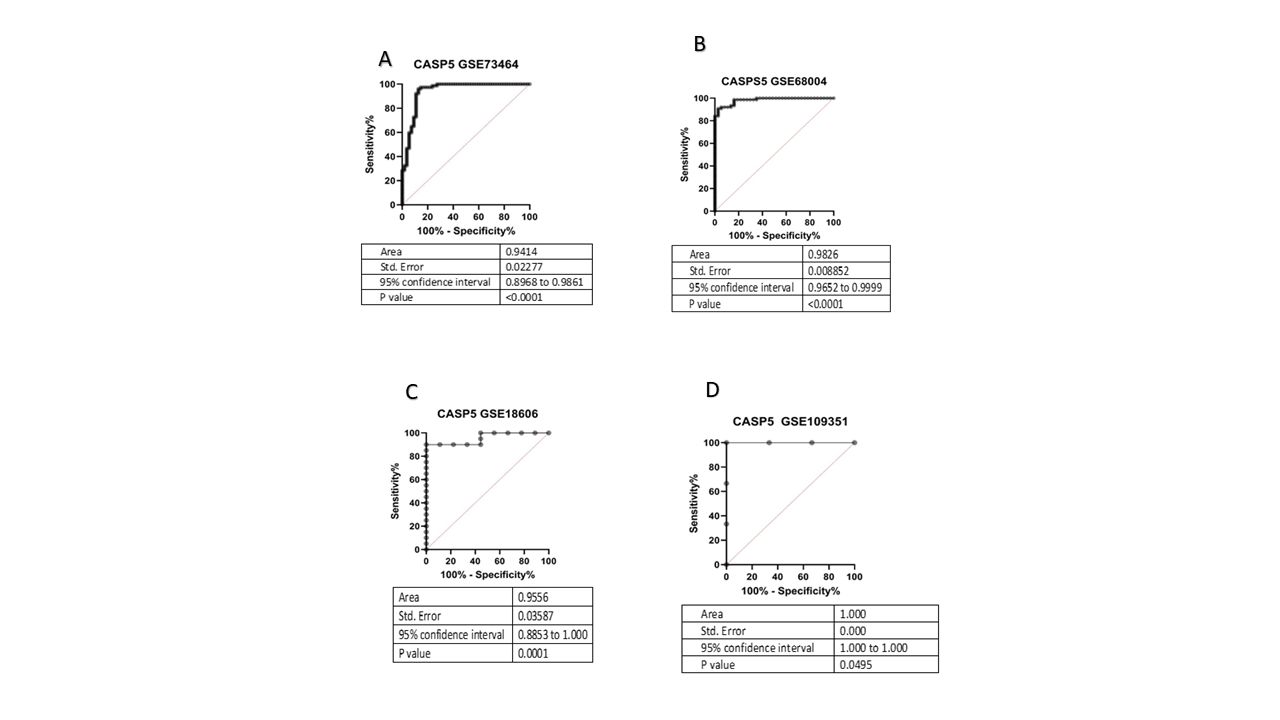


**Supplementary 1.** ROC curve analysis of CASP5 gene diagnosis. Receiver operating characteristic (ROC) curves and area under the curve (AUC) statistics are used to evaluate the capacity to discriminate KD from normal controls with excellent specificity and sensitivity in (A) GSE73464, (B) GSE68004, (C) GSE18606, (D) GSE109351.


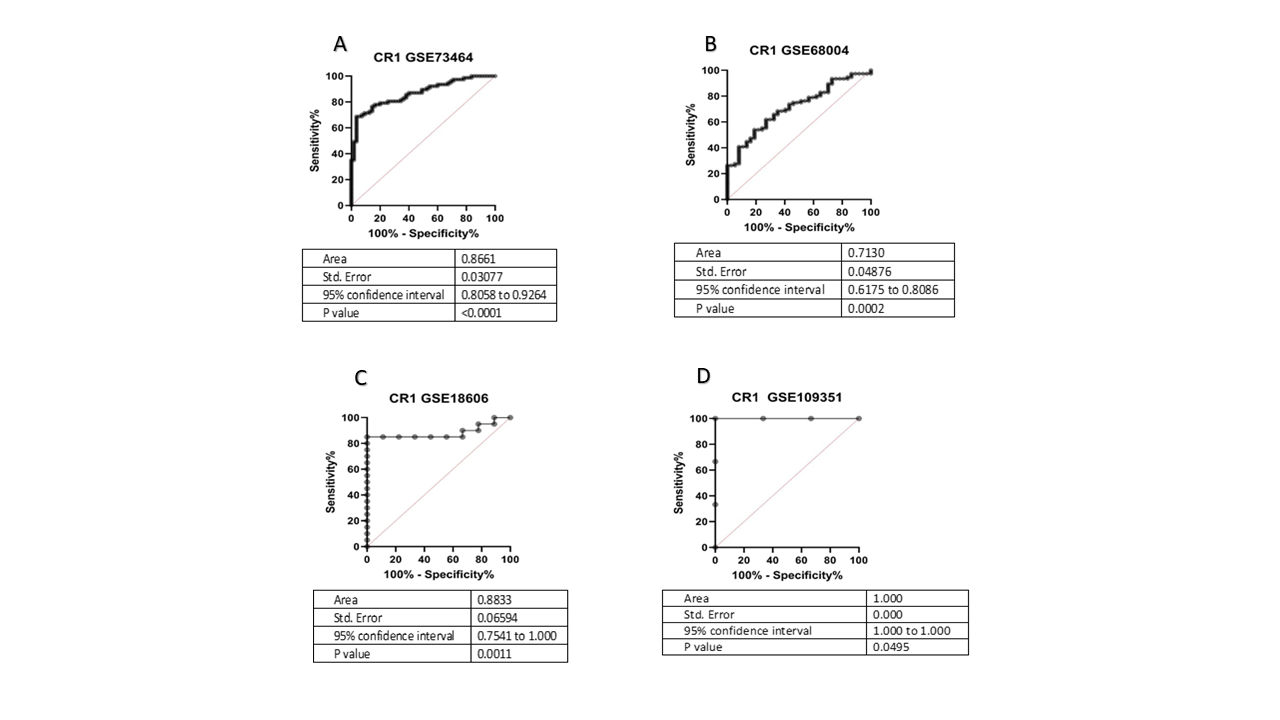


**Supplementary 2.** ROC curve analysis of CR1 gene diagnosis. Receiver operating characteristic (ROC) curves and area under the curve (AUC) statistics are used to evaluate the capacity to discriminate KD from normal controls with excellent specificity and sensitivity in (A) GSE73464, (B) GSE68004, (C) GSE18606, (D) GSE109351.


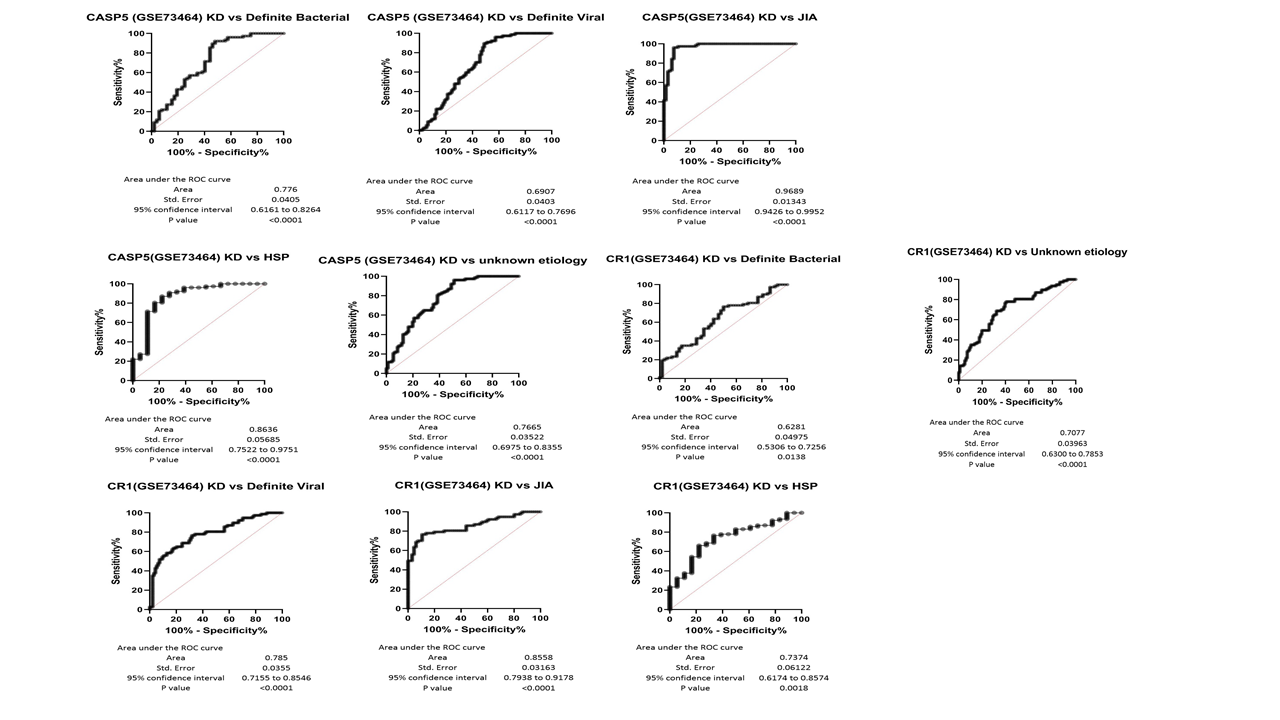


**Supplementary 3.** ROC curve analysis of CASP5 and CR1 genes diagnosis based on GSE73464. Receiver operating characteristic (ROC) curves and area under the curve (AUC) statistics are utilized to determine the capacity to discriminate KD from other febrile conditions with excellent specificity and sensitivity in GSE73464 dataset.
